# Emerging trends in epigenetic and childhood trauma: Bibliometrics and visual analysis

**DOI:** 10.3389/fpsyt.2022.925273

**Published:** 2022-11-15

**Authors:** Yuting Nie, Lulu Wen, Juexian Song, Ningqun Wang, Liyuan Huang, Li Gao, Miao Qu

**Affiliations:** Department of Neurology, Xuanwu Hospital, Capital Medical University, Beijing, China

**Keywords:** CiteSpace, childhood trauma, epigenetics, DNA methylation, visual analysis

## Abstract

**Background:**

The epigenetic study of childhood trauma has become a valuable field. However, the evolution and emerging trends in epigenetics and childhood trauma have not been studied by bibliometric methods.

**Objective:**

This study aims to evaluate status of epigenetic studies in childhood trauma and reveal the research trends based on bibliometrics.

**Methods:**

A total of 1,151 publications related to childhood trauma and epigenetics published between 2000 and 2021 were retrieved from the Web of Science Core Collection (WoSCC). CiteSpace (5.8. R 3) was used to implement bibliometric analysis and visualization.

**Results:**

Since 2010, the number of related publications has expanded quickly. The United States and McGill University are the most influential countries and research institutes, respectively. Elisabeth Binder is a leading researcher in childhood trauma and epigenetic-related research. Biological Psychiatry is probably the most popular journal. In addition, comprehensive keyword analysis revealed that “glucocorticoid receptor,” “brain development,” “epigenetic regulation,” “depression,” “posttraumatic stress disorder,” “maternal care,” “histone acetylation,” “telomere length,” “microRNA,” and “anxiety” reflect the latest research trends in the field. A comprehensive reference analysis demonstrated NR3C1 gene methylation, FKBP5 DNA methylation, BDNF DNA methylation, and KITLG methylation have been hot spots in epigenetic studies in the field of childhood trauma in recent years. Notably, the relationship between childhood adversity and NR3C1 gene methylation levels remains unresolved and requires well-designed studies with control for more confounding factors.

**Conclusion:**

As the best of our knowledge, this is the first bibliometric analysis of the association between childhood trauma and epigenetics. Our analysis of the literature suggests that childhood trauma may induce depression, anxiety, and post-traumatic stress disorder through epigenetic regulation of glucocorticoid receptor expression and brain development. The hypothalamic-pituitary-adrenal axis is the key points of epigenetic research. The current researches focus on NR3C1 gene methylation, FKBP5 DNA methylation, BDNF DNA methylation, and KITLG methylation. These results provide a guiding perspective for the study of epigenetic effects of childhood trauma, and help researchers choose future research directions based on current keywords.

## Introduction

Childhood trauma is of great significance to public health (1). People who experience childhood trauma are not only more likely to develop serious mental health problems, but they also have increased rates of alcohol abuse, suicide, cancer, causing increased mortality and ultimately a huge economic cost to society (2–4). The incidence of child trauma remains high. According to the statistics of 214,157 respondents in 23 states of the United States, 61.55% of them had at least one adverse childhood experience (ACE) and 24.64% had three or more ACE (5). Childhood trauma causes physical and psychiatric complications through long-term effects on mitochondrial function, gut microbiota, hypothalamic-pituitary-adrenal (HPA) axis, inflammation, and epigenetic changes, etc. (6–11). Epigenetic inheritance produces heritable phenotypic changes mainly by physical modification of DNA molecules and chromatin functions, but does not alter DNA sequences. Unlike genetic changes that are difficult to reverse, epigenetics change in response to changes in the surrounding environment and stress. In other words, the environment or stress (especially early childhood trauma) can alter the phenotype of an organism (12). DNA methylation is recognized as the most stable epigenetic modification, followed by histone modifications and non-coding RNA (ncRNA) (13). Early life adversity alters brain structure and development, particularly the amygdala and hippocampus, through epigenetic modifications, leading to stable changes in individual behavior (8, 14). A previous human study showed that childhood trauma affects the development of the neurological and endocrine systems by influencing epigenetic changes and increases the risk of disease and susceptibility to psychiatric disorders (15).

The impact of childhood trauma on DNA methylation is supported by several animal studies. For example, early-life adversity accelerates the aging process through glucocorticoid-induced epigenetic changes and regulates DNA methylation of neurons in mice, which raises arginine pressor, leading to neuroendocrine changes and depression (16, 17). In addition, childhood adversity leads to persistent changes in the state of brain-derived neurotrophic factor (BDNF) DNA methylation, which alters brain development and function, leading to a reduction in brain plasticity (18, 19). Early life adversity alters multiple loci throughout the genome, and the following genes are associated with epigenetic changes associated with childhood adversity: Glucocorticoid receptor gene (NRC31), BDNF, FK506 binding protein 5 (FKBP5), serotonin transporter 5-HTT/SLC6A4. These genes appear to be the most promising therapeutic targets for future research (20–23). Notably, epigenetic marks can be modified by drug targeting, and preclinical studies have considered the possibility of epigenetics as a therapy for post-traumatic stress disorder. For example, valproic acid (VPA), which can act as a histone deacetylase inhibitor, has been shown to reduce oxidative stress and inflammation, and correct disordered neurotransmitters in post-traumatic stress disorder (PTSD) rats (24). Accordingly, targeting the epigenome through specific drug interventions may be a potential option for treating trauma or stress-induced mental illness (25). Given that epigenetics is reversible, late-stage factors such as drugs and the environment can alter the long-lasting effects of trauma and childhood experiences by modifying epigenetics; further research is required in the relationship between trauma and epigenetics (26).

To date, the impact of childhood trauma on epigenetics has not been reported in bibliometrics studies. Through CiteSpace 5.8. R 3 software, this study conducted statistical processing on the literature related to childhood trauma and epigenetic inheritance, and drew a visual network map according to the information of annual publications, institutions, countries, authors and keywords, making the research results quantifiable, more accurate and objective. The countries, authors and institutions were analyzed separately to identify the number of core publications and their collaborative networks. We also evaluated keywords to elaborate hot spots in the field of childhood trauma and epigenetics and show the pace of research development in the field. This paper analyzes the development status, collaboration relationship, research hotspots and research trends of this field in the past 22 years, providing new perspectives and ideas for researchers to conduct research projects, facilitating readers to vividly understand the research status, research hotspots and research trends in this field.

## Materials and methods

CiteSpace^1^ is a visual tool devised by Professor Chen Chaomei to visualize the country, institution, author, keywords, and cited journals, cited authors, and cited references of a document (27).

We entered “childhood trauma” and “epigenetic” as search entries, respectively, spanning 2000-2021 with index = SCI-EXPANDED, SSCI, A&HCI, CPCI-S, CPCI-SSH, ESCI. The language of publication was limited to English. 45.282 articles were found using “childhood trauma” as the search term, 340.646 articles were obtained using “epigenetic” as the search term, and then 1,355 articles were obtained by combining the search results using the conjunction “and.” Subsequent selection of article types as articles and reviews yielded 1,193 articles, and elimination of 42 duplicate articles resulted in 1,151 articles. All data for this study were retrieved from the Web of Science on November 25, 2021. The detailed search terms are illustrated in Supplementary Table 1.

### Data analysis

CiteSpace (5.8. R 3) (See text footnote 1) was performed to analyze data.

The parameters of CiteSpace are as follows: time slicing (2000-2021), years per slice (1), term source (all), node types (one at a time), selection criteria (top 50), pruning (pathfinder/pruning the merged network). The size of the nodes generally predicts the frequency size, while the different colors of the nodes represent different years. Different colored circles from the inside to the outside of the node represent the years 2000 to 2021. In addition, the lines between nodes imply cooperation or co-occurrence or co-referencing relationships. The purple circle indicates centrality, which indicates the strength of the node’s connection role in the network relationship map. Nodes with high centrality are generally considered to be the turning point or hub of a domain and play a strategic undertaking role in the entire internal network, and nodes with centrality greater than 0.1 are usually regarded as key nodes.

## Results

### Distribution characteristics of publications

**FIGURE 1:**
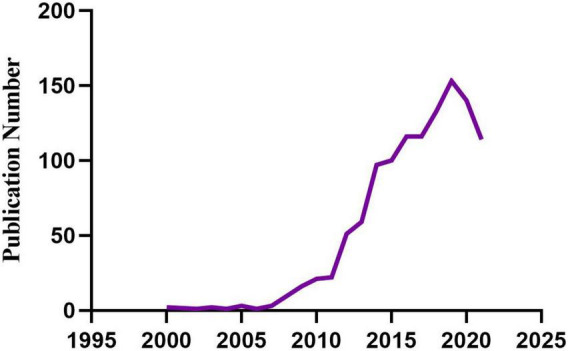
Publication and growth trends in childhood trauma and epigenetics.

We retrieved 1,151 publications meeting the inclusion and exclusion criteria (Figure 1), including 781 articles and 370 reviews. The development trajectory of the last 20 years exhibited two phases: the first phase is the initial phase of development (2000-2009), related research grew very slow, with about 2 publications per year. The second phase is the period of rapid growth (2010-2019), the number of publication outputs increased from 21 in 2010 to 153 in 2019, with an average of 86 publications per year. Although the number of publications declined slightly from 2020 to 2021, overall, the number of relevant publications is still large. In addition, the trend of the numbers shows that there are more and more studies on the relationship between childhood stress and epigenetic modification, and researchers have begun to pay close attention to the effects of childhood trauma on epigenetic modifications.

### Analysis of scientific collaboration network

**FIGURE 2:**
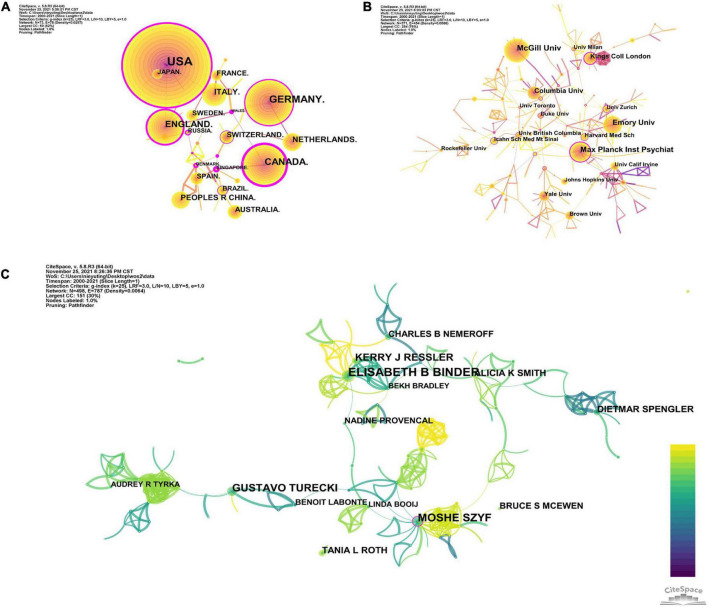
**(A)** Collaboration among countries/regions. **(B)** Collaboration among institutions. **(C)** Collaboration among authors.

Seventy-three major countries or regions have contributed to the field of childhood trauma and epigenetic modification research. Collaborative network between countries/regions was illustrated in Figure 2A, with 73 nodes and 317 link lines. The top ten countries, regions, and authors in terms of number of publications and centrality were listed in Table 1. Higher centrality values indicated more collaboration between that node and other nodes. The top five countries or regions in numbers of papers are the USA, Germany, Canada, England, and Italy in turn. The above results imply a strong interest in studying childhood adversity and epigenetic modifications in these countries. The top five countries in terms of centrality are Wales, Canada, Singapore, Denmark, and New Zealand, demonstrating that these countries play a vital role in studying epigenetics in childhood trauma. Comprehensive analysis shows that the United States (publications: 547, centrality: 0.4) is the most influential country in the field. Although Wales, Singapore, and Denmark do not publish numerous papers, their centricity is more significant than 0.1, indicating their close cooperation with other countries. Unfortunately, no Asian institution was found among the influential countries or regions in the field of childhood trauma and epigenetics. Nonetheless, childhood abuse is still high in Asia, which calls for Asian countries to pay more attention to children suffering from adversity (28).

**TABLE 1:** Top 10 countries/regions, institutions, and authors in terms of publications and centrality.

	Publications			Centrality		
	Ranking	Number	Name	Ranking	Number	Name
Country/Region	1	547	USA	1	0.72	Wales.
	2	180	Germany.	2	0.63	Canada.
	3	147	Canada.	3	0.63	Singapore.
	4	103	England.	4	0.53	Denmark.
	5	63	Italy.	5	0.52	New Zealand.
	6	59	Netherlands.	6	0.44	England.
	7	50	People’s Republic of China.	7	0.4	USA
	8	44	Switzerland.	8	0.31	Germany.
	9	41	Australia.	9	0.31	Norway.
	10	35	France.	10	0.25	Russia.
Institution	1	79	McGill University	1	0.28	Max Planck Institute of Psychiatry
	2	64	Max Planck Institute of Psychiatry	2	0.25	Arq Psychotrauma Expert Group
	3	58	Emory University	3	0.22	Kings College London
	4	42	Columbia University	4	0.19	University of British Columbia
	5	35	Kings College London	5	0.19	University of Pennsylvania
	6	25	Yale University	6	0.18	Boston University
	7	24	Harvard Medical School	7	0.17	Heidelberg University
	8	22	University of British Columbia	8	0.16	University of Bristol
	9	20	Rockefeller University	9	0.16	Glasgow University
	10	19	Duke University	10	0.16	Simon Fraser University
Author	1	27	Elisabeth Binder	1	0.11	Moshe Szyf
	2	22	Moshe Szyf	2	0.06	Gustavo Turecki
	3	20	Gustavo Turecki	3	0.06	Benoit Labonte
	4	17	Kerry J. Ressler	4	0.06	Michael J Meaney
	5	12	Tania Roth	5	0.06	Naguib Mechawar
	6	12	Dietmar Spengler	6	0.05	Elisabeth Binder
	7	11	Alicia K. Smith	7	0.05	Alicia K. Smith
	8	10	Charles B Nemeroff	8	0.05	Nadine Provençal
	9	10	Bruce McEwen	9	0.05	Linda Booij
	10	9	Nadine Provençal	10	0.05	Joan Kaufman

By running CiteSpace software and taking institutions as nodes, a collaborative network of research structures with 371 nodes and 1,042 lines was generated, representing institutions and their cooperative relationships, with a network density of 0.0066, as shown in Figure 2B. The explanation of institutional abbreviations is detailed in Supplementary Table 2. The top 5 institutions contributing the most in this area are McGill University, Max Planck Institute of Psychiatry, Emory University, Columbia University, and King’s College London. McGill University is the most important node, although this institution has a high number of publications (79 publications), it has less collaboration with other institutions. The Max Planck Institute of Psychiatry ranked second with a high centrality, implying an important role in the collaboration relationship.

The author’s network, shown in Figures 2C, Consists of 498 nodes and 925 collaboration lines. In total, 498 authors were included in the author network, of which 235 (47.2%) published only one article. Elisabeth Binder arrived first rank with 27 papers, followed by Moshe Szyf and Gustavo Turecki. Notably, only Moshe Szyf had a centrality greater than 0.1, indicating that he played a central role in the collaboration, while the other authors had a centrality less than 0.1, indicating insufficient collaboration among top scientists. In addition, Elisabeth Binder, Kerry J. Ressler and others formed the academic group, which is located in the center of the network, indicating that the group is in an important position. Meanwhile, there are smaller academic groups distributed around the network map, which are located at periphery, implying that these groups are less influential.

### Keyword co-occurrence and clustering

**FIGURE 3:**
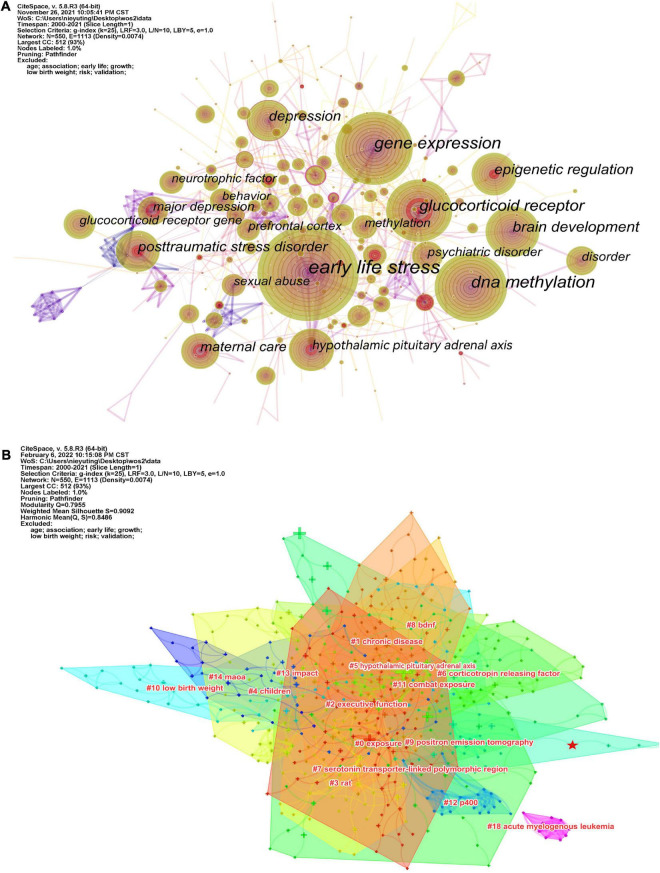
**(A)** Visualization of the keyword co-occurrence network. **(B)** Cluster analysis of keyword networks.

The keyword co-occurrence map reflects the research hotspots. Figure 3A shows a view of the keyword co-occurrence network, resulting in 550 nodes and 3,974 links with a density of 0.0074, which maps the knowledge structure of the study. The size of nodes is positively correlated with the number of keyword occurrences. The top 10 keywords are early life stress (587), DNA methylation (302), gene expression (270), glucocorticoid receptor (205), brain development (139), epigenetic regulation (120), hypothalamic-pituitary-adrenal axis (109), depression (107), posttraumatic stress disorder (102), and maternal care (83). These keywords are shown in Table 2. The keyword “DNA methylation” has become a research hotspot since 2010, appearing in 302 citation studies, which implies that DNA methylation may have excellent research potential. According to the above keywords, the research hotspots of childhood trauma and epigenetic can be summarized in 2 aspects: first, the effects of childhood trauma on DNA methylation, gene expression, glucocorticoid receptor, brain development, epigenetic regulation, and hypothalamic-pituitary-adrenal axis. Second, the mechanism of complications such as depression and post-traumatic stress disorder caused by childhood trauma is related to epigenetic inheritance. Nineteen clusters were generated by log-likelihood ratio, including exposure, chronic disease, executive function, rat, monoamine oxidase A (MAOA), hypothalamic-pituitary-adrenal axis, etc., displayed in Figure 3B. All clusters are interlaced with each other, indicating that the research contents are closely related. Modularity Q (*Q* value) and silhouette value (*S* value) are used to evaluate the scientificity and usability of visual knowledge graphs. In this study, the modularity *Q* = 0.7955, when *Q* > 0.3, indicates that the network clustering structure is significant; the silhouette value = 0.9092, when *S* > 0.5, indicates that the clustering results are highly reliable.

**TABLE 2:** The top 10 keywords.

Ranking	Frequency	Centrality	Years	Keywords
1	587 (10%)	0.01	2007	Early life stress
2	302 (5.0%)	0	2010	DNA methylation
3	270 (4.5%)	0.01	2005	Gene expression
4	205 (3.5%)	0.09	2005	Glucocorticoid receptor
5	139 (2.4%)	0.01	2009	Brain development
6	120 (2.0%)	0.01	2010	Epigenetic regulation
7	109 (1.8%)	0.03	2007	Hypothalamic-pituitary-adrenal axis
8	107 (1.8%)	0.12	2009	Depression
9	102 (1.7%)	0.04	2007	Posttraumatic stress disorder
10	83 (1.4%)	0.06	2009	Maternal care

### Keyword burst detection analysis

**FIGURE 4:**
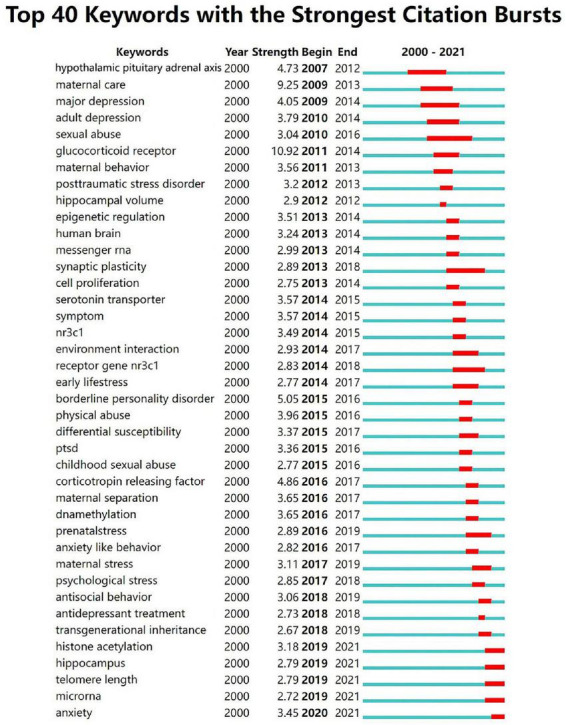
Top 40 keywords with the strongest citation bursts.

Figure 4 shows the top 40 keywords with the strongest citation bursts. The “glucocorticoid receptor” was the most powerful keyword in the field between 2011 and 2014 (strength: 10.92), followed by maternal care (strength: 9.25), borderline personality disorder (strength: 5.05). The 2019-2021 outbreak keywords include “histone acetylation,” “hippocampus,” “telomere length,” “microRNA,” “anxiety,” which reflect the latest research trends. Recent studies have shown that the effects of childhood trauma on “histone acetylation,” “hippocampus,” telomere length, and “microRNA” are becoming more and more important. Hypothalamic-pituitary-adrenal axis (2007-2012), major depression (2009-2014), sexual abuse (2010-2016), synaptic plasticity (2013-2018), glucocorticoid receptor gene NR3C1 (2014-2018) have been studied for a long time, indicating that these research directions are of significant value and scientists have invested a lot of time and funding in these directions.

### Co-citation by author and journal

**FIGURE 5:**
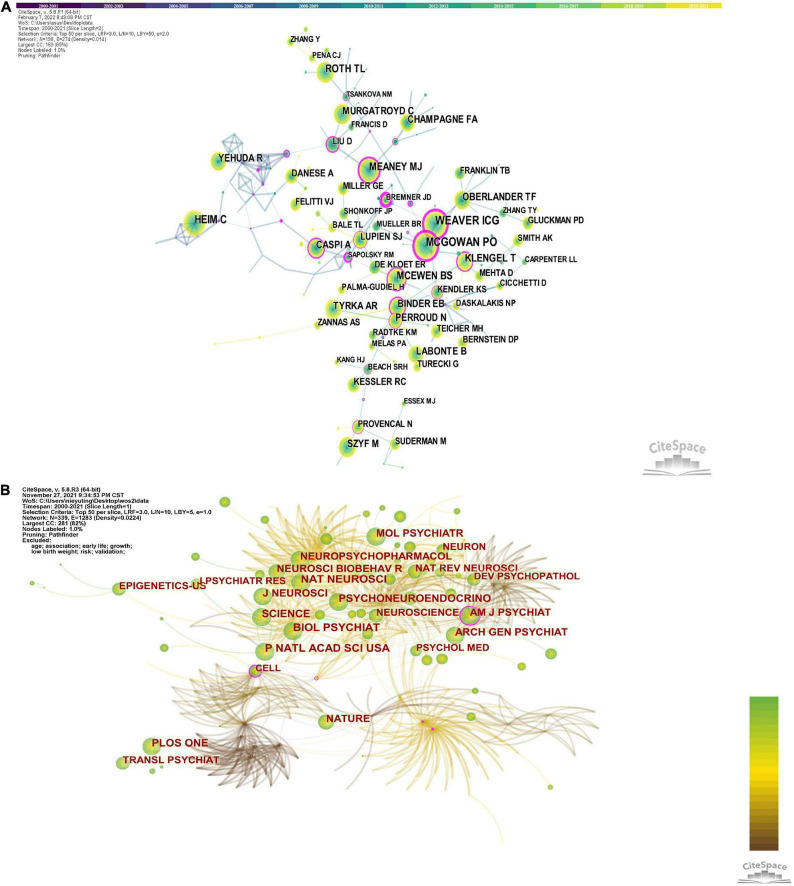
**(A)** Networks of co-reference of author. **(B)** Network of co-reference of journal.

Figure 5A shows the network of co-reference of the author and the journal. The authors most frequently cited are Ian Weaver (461) and Patrick McGowan (461), followed by Christine Heim (352), Torsten Klengel (261), Bruce McEwen (258), and Theodore Roth (254). Furthermore, the centrality of Ian Weaver, Patrick McGowan and Christine Heim are 0.83, 0.7, 0.64, respectively.

The network diagram generated by journal co-citations is shown in Figure 5B, and the explanation of journal abbreviations is detailed in Supplementary Table 3. Table 3 presents the top 10 co-cited journals for epigenetic studies in childhood abuse. The top three journals in terms of co-citations were Biological Psychiatry (publications: 911), followed by Proceedings of the National Academy of Sciences of the United States of America (publications: 834), Nature Neuroscience (publications: 825), and PLoS One (publications: 794). The journal with a median centrality ≥0.1 is the American Journal of Psychiatry (centrality: 0.29). It is worth noting that Science has the highest impact factor (IF: 47.728) and is ranked 6th among co-cited journals. Thus, it plays an essential role in this research area, followed by Nature Neuroscience with an IF of 24.884. The average IF of the top 10 journals is 15.35, which means that these studies are very reliable and of very high quality. Biological Psychiatry is probably the most popular journal regarding the number of publications and impact factors.

**TABLE 3:** The top 10 co-cited Journal.

Ranking	Times cited	Centrality	Years	Journal	Five-year average IF
1	911	0.05	2005	Biological Psychiatry	14.102
2	834	0.04	2000	Proceedings of the National Academy of Sciences of the United States of America	12.291
3	825	0.01	2005	Nature Neuroscience	25.874
4	794	0.03	2009	PLoS One	3.788
5	707	0.01	2005	Psychoneuroendocrinology	5.663
6	675	0.05	2000	Science	51.434
7	663	0.02	2005	Neuropsychopharmacology	8.21
8	655	0.01	2005	Journal of Neuroscience	6.993
9	644	0.02	2005	Molecular Psychiatry	14.806
10	606	0.29	2002	American Journal of Psychiatry	17.825

IF, Impact Factor.

### Analysis of co-citation references

**FIGURE 6:**
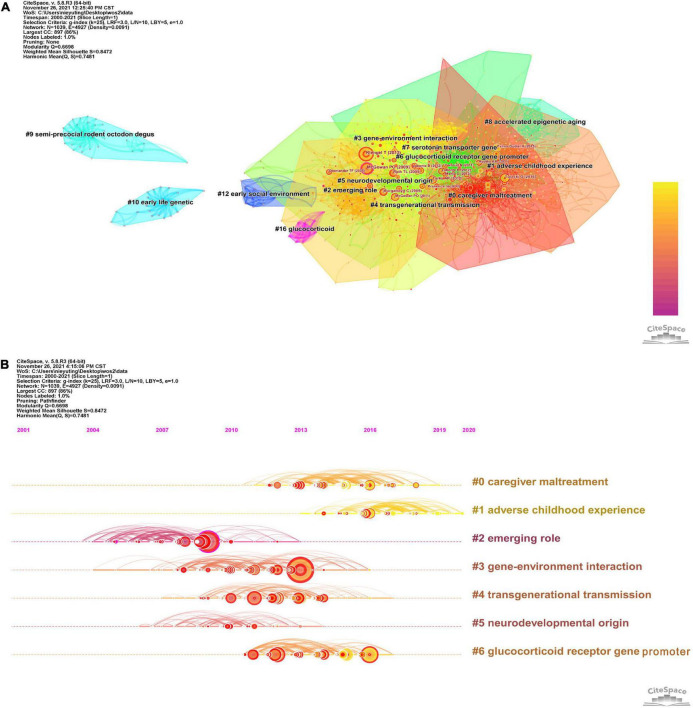
**(A)** The clustering of the reference co-citation network. **(B)** Timeline view based on co-citation analysis of reference in childhood trauma-epigenetics from 2000 to 2021.

When two or more papers are cited in one paper at the same time, the two (or more) papers constitute a co-citation relationship. The clustering and key nodes in the co-citation network can reveal the literature that plays a key role in the evolution of the research frontier in the field. The clustering of the reference co-citation network by the LLR algorithm has been divided into 17 clusters (*Q* = 0.6698, *S* = 0.8472), which are shown in Figure 6A. The largest seven groups of references in the co-citation network were selected, and the details are shown in Table 4. The visual timeline contains 1,039 nodes and 4,927 links, representing cited references and their co-citation relationships. Figure 6B shows a timeline of epigenetic research in childhood trauma based on the CiteSpace software. Gene-environment interaction (cluster # 3) has the most significant node, node size represents the number of times of citation. The study of Klengel et al. showed the largest node (48 citations). The study illustrated the interaction between the FKBP5 gene and long-range enhancers, resulting in differential transcriptional activation of FKBP5 and an increased risk of childhood psychosis (29). Based on literature co-citation clustering, it can be hypothesized that childhood trauma affects neurodevelopment, glucocorticoid receptor gene promoters, and induces psychiatric disorders through epigenetic modulation and that this inheritance produces transgenerational transmission.

**TABLE 4:** The largest 7 clusters of references in the co-citation network.

Cluster ID	Size	Silhouette	Mean (Year)	Label (LLR)
0	145	0.806	2014	Caregiver maltreatment
1	132	0.782	2017	Adverse childhood experience
2	120	0.89	2007	Emerging role
3	105	0.862	2010	Gene-environment interaction
4	97	0.731	2012	Transgenerational transmission
5	61	0.878	2009	Neurodevelopmental origin
6	61	0.875	2013	Glucocorticoid receptor gene promoter

### Analysis of reference burst detection

**FIGURE 7:**
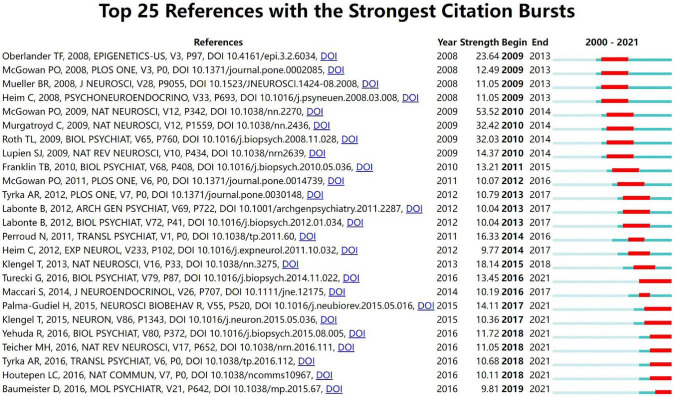
Top 25 reference with the strongest citation burst in childhood trauma-epigenetics research.

Figure 7 shows 25 references with strong citation outbursts. The duration of the burst is shown in red. The strongest burst (strength: 53.52) appeared in a 2010 article (30). Five recent groundbreaking papers highlighted new trends in trauma and epigenetics research in 2021 (7, 31–34).

### Author and keywords time zone atlas

**FIGURE 8:**
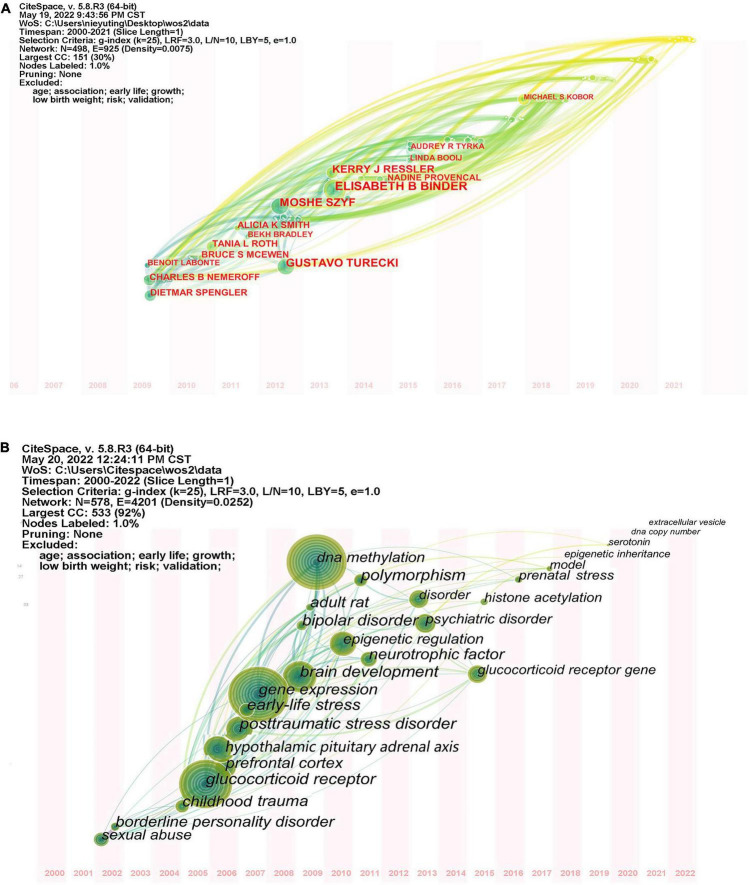
**(A)** Author time zone atlas. **(B)** Keywords time zone atlas.

Citespace author time zone atlas visualizes authors by year of publications (Figure 8A), Pruning network is “Pathfinder” + “Pruning the tender network” + “Pruning the merged networks.” From 2009 to 2011, Dietmar Spengler, Charles B. Nemeroff and Benoit Labonte were the main authors in this field. Between 2012 and 2013, there were four prominent contributors in this field, namely Gustavo Turecki, Moshe Szyf, Elisabeth Binder, Kerry J. Ressler. These authors have been still in contact with many authors from recent years, indicating that the authors will continue to pay attention to research in this area. Since 2014, there has been an influx of new authors, but most have not conducted in-depth, sustained research.

The keyword time zone atlas function can clearly show the development process of this research field in each time period, and vividly show the evolution direction of research focus (Figure 8B). The time zone view can be analyzed from two periods: before 2005, there was little research in this field, which did not attract wide attention of researchers, so there were only a few hot keywords; since 2006, glucocorticoid receptor, DNA methylation, gene expression, brain development, epigenetic regulation, hypothalamic pituitary adrenal axis, depression, and posttraumatic stress disorder gradually emerged and achieved rapid development. The large diameter of these nodes indicates that the research results in this period played a key role in the field. There are many connections between these nodes and keywords appearing in recent years, suggesting that research in this period is closely related to research in recent years.

## Discussion

To the best of our knowledge, this is the first bibliometric analysis of epigenetic studies in the field of childhood trauma. Through analyzing 1,151 publications from WoSCC, we provide a comprehensive overview of global hotspots and trends in childhood trauma and epigenetics research over the past 22 years. Our analysis shows a rapid increase in publications since 2010 and a lack of international scientific collaboration in this field. USA, McGill University, Elisabeth Binder are the most influential country, institute, and scholar. The most popular journal is Biological Psychiatry. In addition, a comprehensive keyword analysis revealed that glucocorticoid receptor, brain development, epigenetic regulation, hypothalamic-pituitary-adrenal axis, depression, posttraumatic stress disorder, maternal care, histone acetylation, hippocampus, telomere length, microRNA, and anxiety reflect the latest research trends in the field. Combining the reference literature analysis results, we found that NR3C1 gene methylation, FKBP5 DNA methylation, BDNF DNA methylation, KITLG methylation, and inflammatory phenotypes are popular points for epigenetic studies in the field of childhood trauma citation. Therefore, our findings provide hot spots and research trends regarding epigenetic studies in the field of childhood trauma in order to facilitate more in-depth studies in this field.

A visual analysis of the distribution of countries and institutions shows that the United States and Germany are the leading countries in epigenetic research on childhood trauma. The top 5 institutions are, in order, McGill University, Max Planck Institute of Psychiatry, Emory University, Columbia University, and King’s College London. We found very little collaboration between these institutions, and therefore we strongly expect these institutions to collaborate and communicate with each other to facilitate the development of epigenetic research in the field of childhood trauma.

Elisabeth Binder ranks first in terms of author contributions and co-authors, with 27 published papers. Elisabeth Binder’s team concentrated on the methylation of the FKBP5 gene, PTSD, and childhood trauma. Childhood trauma can affect DNA methylation, and in one frequently cited cohort study, carriers of the FKBP5 risk allele had a substantially enhanced risk of developing PTSD after experiencing early trauma (29). Furthermore, trauma experience can also have intergenerational effect on FKBP5 methylation (34). This literature shows that Elisabeth Binder has made some breakthroughs in the study of FKBP5 gene methylation and PTSD in childhood trauma. Despite the high number of publications and citations in the field of study and the high quality of the authors’ research, collaboration with other researchers is rare. In addition, with a network centrality <0.1, the impact is still not high enough. Therefore, we recommend more collaborations in the future.

According to co-occurrence keyword analysis, we have identified some of the most critical hot spots in this field in the past two decades. Comprehensive keyword analysis revealed glucocorticoid receptor, brain development, epigenetic regulation, hypothalamic-pituitary-adrenal axis, depression, posttraumatic stress disorder, maternal care, histone acetylation, hippocampus, telomere length, microRNA, and anxiety reflect the latest research trends in the field. In addition, keyword clustering analysis shows that scientists are not limited to the effects of childhood trauma on neuropsychiatric disorders, but are beginning to focus on the effects of childhood trauma on systemic health and chronic diseases such as diabetes, respiratory diseases, and heart disease. Childhood trauma increases the risk of mental disorders and chronic diseases such as diabetes and coronary heart disease, and affects aging through genetic modifications (35). An epidemiological study in the United States, adjusting for multiple confounders, found that childhood trauma increased the prevalence of chronic diseases such as chronic obstructive pulmonary disease, coronary heart disease, asthma, cancer, stroke, and diabetes (36). Another large-scale study in the Netherlands found that childhood trauma was a risk factor for digestive system, musculoskeletal diseases and respiratory system diseases (37).

Journal analysis can reflect the influential journals in the research field and help authors’ submissions. The total impact factors of the top 10 journals ranged from 3 to 51, and six journals had a five-year average IF >10.00, suggesting that the studies were very reliable and of high quality. The journal Biological Psychiatry has a particular interest in childhood trauma and epigenetic research. It’s worth noting that the most frequently cited article in Biological Psychiatry, Science, Nature Neuroscience, Proceedings of the National Academy of Sciences of the United States of America, and PLoS One is “Epigenetic regulation of the glucocorticoid receptor in human brain associates with childhood abuse” (30). This paper was the first to find that the expression of NR3C1 gene in the hippocampus of human samples of suicide victims who experienced childhood abuse is lower than that of dead victims who did not experience childhood abuse, a key finding in this field.

Frequent co-citation references focused on the effects of childhood adversity on methylation of the NR3C1 promoter methylation, FKBP5 DNA methylation, and BDNF DNA methylation. A prospective study of 99 samples found that childhood abuse and parental neglect increased methylation of the NR3C1 promoter; there was also a positive association between the severity of child abuse and methylation of the NR3C1 promoter (15, 38). On the contrary, one study showed that suicide victims experiencing childhood abuse had decreased expression of the NR3C1 gene in the hippocampus compared with those without abuse (30). Therefore, more research is needed to further confirm these links. In addition, carriers of the FKBP5 risk allele may have increased glucocorticoid sensitivity through epigenetic mechanisms after early trauma (29). For BDNF DNA methylation, a clinical study has proven that early abuse leads to sustained enhancement of BDNF gene expression in the prefrontal cortex in adults, and the DNA methylation inhibitors could reverse these altered epigenetic marks and gene expression (39). Moreover, alterations in the epigenetic machinery of childhood trauma or abuse involve not only a single gene promoter, but also multiple genes, multiple promoters, and even a wide range of effects across the genome (40–42). A comprehensive literature analysis shows that childhood trauma can cause changes in several genes and promoters, with methylation of the NR3C1, FKBP5, and BDNF as hotspot genes.

The latest five burst papers represent new trends in pediatric trauma and epigenetic research from 2018-2021, are highly regarded and represent the core of current research. For instance, one 10-year longitudinal study revealed that parents who experienced the Holocaust produced changes in FKBP5 methylation and that such changes were also observed in their offspring. This result demonstrates that psychological trauma may produce intergenerational effects by modulating epigenetic changes (34). Teicher reviewed the effects of early trauma-induced epigenetic modifications on brain development, structure and function (32). Contrary to a previous study (38), Tyrka recruited 340 subjects and found that childhood adversity was associated with lower levels of NR3C1 gene methylation, and lower cortisol response (33). Houtepen identified a locus in the Kit ligand gene (KITLG; cG27512205) which associated with cortisol stress response using genome-wide analysis, and shedding light on the neurobiological pathways behind individual’s vulnerability to stress (31). Saygideger conducted a meta-analysis and revealed that childhood trauma induces asthma or other chronic inflammatory diseases by influencing genes such as NR3C1 and FKBP5, which mediate immune inflammation (43). The above studies suggest that FKBP5 methylation, NR3C1 methylation, KITLG methylation, and inflammatory phenotypes have been the focus of epigenetic studies in the field of childhood trauma in recent years. Notably, the relationship between childhood adversity and NR3C1 gene methylation levels remains unresolved and requires well-designed studies controlling more confounding factors.

Our study has some strengths and limitations. We conducted a comprehensive analysis of nearly all publications on epigenetics and childhood trauma, covering studies of DNA methylation, histone modifications, chromatin remodeling, and regulation of noncoding RNA. This study reveals hot spots and frontiers of research on childhood trauma and epigenetic modifications, and may facilitate more in-depth studies in this field. Due to the limited data types processed by the software itself, this study only selected data sources from the WoSCC database, which may exclude some research results from other databases. In the future, the research and development of multi-database document information synchronization conversion function will further enrich the data sources. In addition, due to the existence of multiple synonyms such as childhood trauma, sexual abuse, depression, and major depression, there may be some overlap in keyword analysis. Finally, these results were not compared with contemporaneous epigenetic studies.

## Conclusion

This study analyzed the hot spots and frontiers of research on childhood trauma and epigenetic modifications through CiteSpace. At present, researchers in this field focus on the mechanism of neuropsychiatric disorders caused by childhood trauma and its mechanism. The research progress of NR3C1, FKBP5, BDNF, KITLG and other genes will provide a solid theoretical basis for the improvement of mental disorders in children after trauma. Epigenetic changes caused by childhood trauma can lead to a range of neuropsychiatric disorders and even affect inheritance across generations. However, attention to childhood trauma is still limited in developing countries, especially in Asia. We look forward to enhanced cooperation among the countries and institutes to promote faster development in this field. Overall, our findings provide valuable information for defining new ideas and shaping future research directions.

## Data availability statement

The original contributions presented in this study are included in the article/Supplementary material, further inquiries can be directed to the corresponding author/s.

## Author contributions

MQ and LG designed this research and reviewed the manuscript. YN drafted the manuscript, as well as the acquisition, analysis, and interpretation of data. LW, JS, NW, and LH contributed to literature search and interpretation of the results. All authors have read and approved the manuscript.

## Footnotes

1
http://cluster.cis.drexel.edu/∼cchen/citespace/
