# Supplementary material for: Emerging trends in epigenetic and childhood trauma: Bibliometrics and visual analysis
Source: Front Psychiatry. 2022 Nov 15;13:925273. doi: 10.3389/fpsyt.2022.925273 (PMC9705591; doi:10.3389/fpsyt.2022.925273)
Supplement: Supplementary file 1 [file Table_1.DOCX]

Supplementary table 1 | search strategy and results

| #1 | 45.282 | TS=("early life stress events " OR " early life stress" OR " early adversity " OR " childhood trauma" OR " stress in early life " OR " adverse childhood experiences " OR " adversity in early life " OR " Childhood maltreatment " OR " childhood abuse" OR " childhood trauma" OR " childhood stress" OR " Early Adverse Life Events" OR " Childhood adversity" OR "emotional abuse "OR"emotional maltreatment"OR"physical abuse " OR " physical maltreatment" OR "severe physical abuse" OR "bodily maltreatment" OR "sexual maltreat" OR "sexual abuse" OR "sex abuse" OR "ELA" OR "early life adversity" OR " adverse childhood " ) |
| --- | --- | --- |
| #2 | 340.654 | TS= ("epigenetics " OR "epigenetic " OR "epigenetic inheritance" OR "epigenetic heredity" OR "epigenetic effects" OR "apparent genetics" OR "epigenetic effect " OR "methylation " OR "methylation patterns " OR "methylating" OR "methylate " OR "methylation profile" OR "gene methylation" OR "aberrant methylation" OR "demethylation " OR "DNA methylation" OR "histone modification " OR "histone modifications " OR "modification of histones" OR "histone modulation" OR "genome protein modification" OR "histone modified " OR "chromatin remodeling" OR "chromatin remodel " OR "chromosome remodeling " OR "chromatin remolding " OR "chromatin remodelling" OR "chromatin reshaping " OR " non-coding RNA " OR "non-coding RNAs " OR "long non-coding RNA " OR "none-coding RNA " OR "non-protein coding RNA " OR "small non-coding RNA" OR "non-coding ribonucleic acid" OR "non-encoding RNAs " OR "miRNA" OR " genomic imprinting" OR "genome imprinting" OR "genetic imprinting " OR "imprinted gene" OR "genomics imprinting " OR "gene imprinting " OR "histone acetylation" OR "histone deacetylation" OR "histon acetylation " OR "histidine acetylation" OR "histon acetylization " OR "histone acetyltransferase " OR "acetylization of histone" ) |
| #3 | 1151 | #1 and #2 |
|  |  | Index =SCI-EXPANDED, SSCI, A&HCI, CPCI-S, CPCI-SSH, ESCI |
