# Supplementary material for: Emerging trends in epigenetic and childhood trauma: Bibliometrics and visual analysis
Source: Front Psychiatry. 2022 Nov 15;13:925273. doi: 10.3389/fpsyt.2022.925273 (PMC9705591; doi:10.3389/fpsyt.2022.925273)
Supplement: Supplementary file 2 [file Table_2.DOCX]

Supplementary Table 2 The explanation of institutional abbreviations

| Abbreviations | Full name |
| --- | --- |
| Emory Univ | Emory University |
| Columbia Univ | Columbia University |
| Kings Coll London | Kings College London |
| Yale Univ | Yale University |
| Harvard Med Sch | Harvard Medical School |
| Univ British Columbia | University of British Columbia |
| Rockefeller Univ | Rockefeller University |
| Duke Univ | Duke University |
| Univ Calif Irvine | University of California, Irvine |
| Univ Toronto | University of Toronto |
| Brown Univ | Brown University |
| Univ Milan | The University of Milan |
| Icahn Sch Med Mt Sinai | the Icahn School of Medicine at Mount Sinai |
| Univ Zurich | University of Zurich |
| Johns Hopkins Univ | Johns Hopkins University |
