# Supplementary material for: Emerging trends in epigenetic and childhood trauma: Bibliometrics and visual analysis
Source: Front Psychiatry. 2022 Nov 15;13:925273. doi: 10.3389/fpsyt.2022.925273 (PMC9705591; doi:10.3389/fpsyt.2022.925273)
Supplement: Supplementary file 3 [file Table_3.DOCX]

Supplementary Table 3 The explanation of journal abbreviations

| Abbreviations | Full name |
| --- | --- |
| BIOL PSYCHIAT | Biological Psychiatry |
| P NATL ACAD SCI USA | Proceedings of the National Academy of Sciences of the United States of America |
| NAT NEUROSCI | Nature Neuroscience |
| PLOS ONE | PLoS One |
| PSYCHONEUROENDOCRINO | Psychoneuroendocrinology |
| SCIENCE | Science |
| NEUROPSYCHOPHARMACOL | Neuropsychopharmacology |
| J NEUROSCI | Journal of Neuroscience |
| MOL PSYCHIATR | Molecular Psychiatry |
| AM J PSYCHIAT | American Journal of Psychiatry |
| ARCH GEN PSYCHIAT | Archives of General Psychiatry |
| NEUROSCI BIOBEHAV R | Neuroscience and Biobehavioral Reviews |
| NATURE | Nature |
| TRANSL PSYCHIAT | Translational Psychiatry |
| NAT REV NEUROSCI | Nature Reviews Neuroscience |
| EPIGENETICS-US | Epigenetics |
| NEUROSCIENCE | Neuroscience |
| J PSYCHIATR RES | Journal of Psychiatric Research |
| DEV PSYCHOPATHOL | Development and Psychopathology |
| NEURON | Neuron |
| PSYCHOL MED | Psychological Medicine |
